# **β**-Arrestin1/2 are essential for embryonic lymphatic vessel development

**DOI:** 10.1172/jci.insight.198032

**Published:** 2026-03-26

**Authors:** Yanna Tian, D. Stephen Serafin, Monserrat Avila-Zozaya, Alyssa M. Tauro, Natalie M. Torres-Valle, Bryan M. Kistner, Danielle M. Dy, Elizabeth S. Douglas, Kathleen M. Caron

**Affiliations:** Department of Cell Biology and Physiology, the University of North Carolina at Chapel Hill, Chapel Hill, North Carolina, USA.

**Keywords:** Development, Vascular biology, Endothelial cells, G protein-coupled receptors, Lymph

## Abstract

β-Arrestins are ubiquitously expressed cytosolic adaptor proteins that regulate GPCR-dependent and -independent pathways essential for numerous physiological functions. This study investigated the role of β-arrestin1/2 in embryonic lymphatic vessel development and survival by generating and characterizing mice with lymphatic tamoxifen-inducible loss of the genes encoding β-arrestin1/2 (*Arrb1/2*^ΔiLEC^). At E15.5, *Arrb1/2*^ΔiLEC^ embryos exhibited profound hydrops fetalis and increased embryonic mortality compared with control *Arrb1/2^fl/fl^* embryos. Edematous *Arrb1/2*^ΔiLEC^ embryos, which were more often represented by the female sex, showed growth restriction and decreased lymphatic endothelial cell (LEC) proliferation in the jugular lymphatic sac compared with controls. In vitro knockdown of β-arrestin1 in LECs increased proliferation and increased activation of AKT, while knockdown of β-arrestin2 decreased proliferation and decreased activation of both ERK and CREB. *Arrb1/2*^ΔiLEC^ embryos also exhibited dilated dermal lymphatics with decreased continuous VE-cadherin adherens junctions compared with controls. These results were recapitulated in vitro in β-arrestin1/2 knockdown human LECs, which showed a decrease in membrane VE-cadherin and β-catenin levels, in addition to prevention of adrenomedullin-induced linearization of VE-cadherin at endothelial cell–cell junctions. Collectively, these results demonstrate that loss of β-arrestin1/2 in lymphatics causes hydrops fetalis, midgestational growth arrest, and embryonic demise associated with reduced LEC proliferation and disrupted VE-cadherin adherens junctions.

## Abstract 2

Lymphatic dysfunction can lead to fetal loss due to interstitial edema. Loss of β-arrestin1/2 in lymphatics disrupts lymphatic development and causes embryonic mid-gestational arrest.

## Introduction

β-Arrestins (β-arrestin1, gene *ARRB1*, and β-arrestin2, gene *ARRB2*, also called arrestin 2 and 3, respectively) are multifunctional, ubiquitously expressed intracellular adaptor proteins that were first discovered for their roles in regulating G protein–coupled receptor (GPCR) desensitization and internalization (1–3). Agonist stimulation of GPCRs results in receptor phosphorylation by GPCR kinases (GRKs), which in turn initiates β-arrestin recruitment to facilitate receptor desensitization (4, 5). Recent studies have expanded the multidimensional roles of β-arrestins as scaffolding proteins that affect many facets of cellular signaling (6, 7). For example, β-arrestins serve as requisite adaptor proteins for E3 ubiquitination of GPCRs during internalization and recycling. Further, β-arrestin recruitment to GPCRs can simultaneously trigger a distinct set of subcellular compartmentalized downstream signals, such as activation of the MAPK/ERK signaling pathway, in a process called β-arrestin–biased signaling (8, 9). In addition to regulation of GPCRs, β-arrestins are also known to regulate various non-GPCR signaling pathways (10).

β-Arrestin1 and -2 share 78% sequence similarity, which conveys both distinct and overlapping functions (11, 12). Current studies suggest β-arrestins exhibit different affinities to various classes of GPCRs and display distinct conformational changes upon recruitment (13). For example, β-arrestin2 is generally considered to have a higher affinity for class A GPCRs compared with β-arrestin1 (13). In some contexts, β-arrestin2 shows a greater ability to act as a scaffold and sustain MAPK signaling, whereas β-arrestin1 is more involved in nuclear signaling events. Of note, although mice that lack both genes encoding for β-arrestin1 and -2 die during the neonatal period, mice that lack either β-arrestin1 or β-arrestin2 do not exhibit overt phenotypes, which suggests that the β-arrestins can functionally compensate for the loss of the other (14, 15).

Both β-arrestin1 and -2 have been intensely studied in many tissues and cells. In the blood vascular system, β-arrestin–mediated signaling is critical for angiogenesis, vasodilation, cell proliferation, migration, and immune function (16). Both β-arrestin1 and -2 regulate VEGFR-mediated endothelial cell functions (17, 18), and β-arrestin2 directly interacts with vascular endothelial cadherin (VE-cadherin) to regulate its internalization and increase vessel permeability (18–20). However, the role of β-arrestin1 and -2 in the lymphatic vasculature has yet to be defined.

Lymphatic vessels are critical for maintaining fluid homeostasis, immune cell trafficking, and chylomicron absorption in the small intestine. During early mouse development, around E9.5–E10.5, most lymphatic endothelial cells (LECs) arise from venous endothelial progenitors through expression and activation of lymphatic-cell fate transcription factors, such as prospero-related homeobox 1 (Prox1) (21). Between E10.5 and E15.5, these nascent LECs migrate away from the jugular vein to form a jugular lymphatic sac (JLS), which continues to proliferate into a primary lymphatic plexus (22). From E15.5 to the early postnatal period, LECs further differentiate into distinct lymphatic beds comprising lymphatic capillaries, pre-collectors, and lymphatic collector vessels. The scaled migration of the lymphatic vasculature depends on continuous maintenance of impermeable, zipper-like VE-cadherin adherens junctions until E16.5, after which the junctions transform into loose, button-like junctions to mediate fluid uptake (23–25). Developmental abnormalities in lymphatic vessel growth can result in a condition called hydrops fetalis, which is almost always associated with embryonic lethality. Dysfunction of lymphatics can lead to lymphatic-associated and sometimes life-threatening conditions, such as lymphedema, which is characterized by the accumulation of interstitial fluid in affected tissues (26). In fact, an estimated 250 million individuals worldwide have lymphatic-associated disorders, and yet no effective treatments are available. Thus, efforts to better understand the mechanisms of lymphatic growth, with a focus on therapeutically tractable factors like GPCRs, remain a high priority.

GPCRs are central regulators of lymphangiogenesis and represent promising therapeutic targets (27). Several GPCR pathways, such as calcitonin receptor–like receptor (CALCRL) signaling activated by adrenomedullin (AM) (28–31), sphingosine-1-phosphate receptor-1 (S1PR1) (32, 33), apelin receptor (APLNR) (34–36), GPRC5B (37), and the atypical chemokine receptor ACKR3 (38, 39), are essential for lymphatic vessel development, remodeling, and function, with disruption of these pathways leading to severe lymphatic defects, embryonic edema, or pathological remodeling. As key mediators of GPCR desensitization, trafficking, and biased signaling, the role of β-arrestin1/2 in lymphatic vessels remains largely unexplored. Defining how β-arrestin1/2 integrate GPCR signaling is therefore critical to understanding the molecular control of lymphatic vessel development and disease.

In this study, we investigated the role of β-arrestin1 and β-arrestin2 in embryonic and lymphatic development in vivo through generation of a conditional lymphatic-specific double β-arrestin1 and β-arrestin2 KO mouse, with cell biological findings complemented by mechanistic in vitro studies using human primary LECs. The results support essential functions for both β-arrestin1 and -2 in lymphatic vessel development and in regulating LEC proliferation and VE-cadherin adherens junctional assembly.

## Results

### Arrb1/2^ΔiLEC^ embryos exhibit hydrops fetalis, increased lethality, and growth restriction.

**Figure 1:**
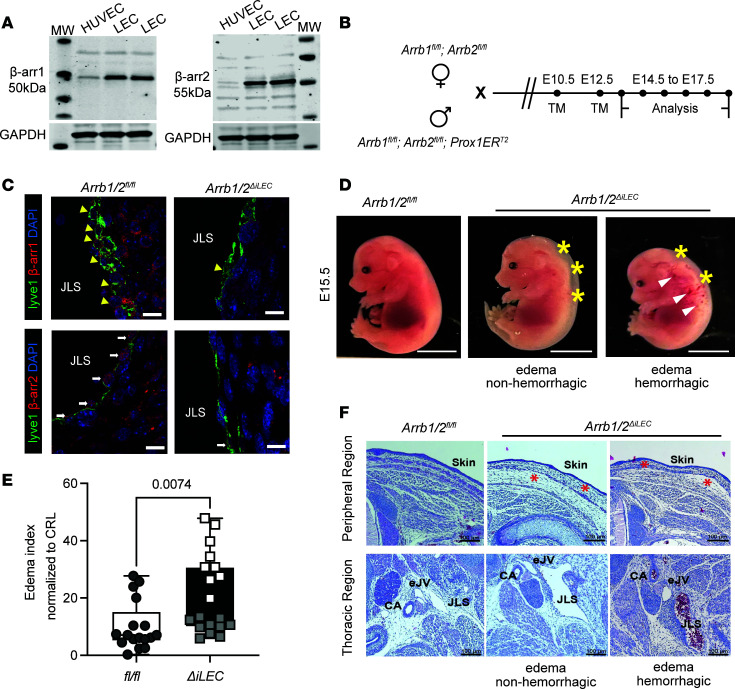
Hydrops fetalis in *Arrb1/2^ΔiLEC^* embryos compared with *Arrb1/2^fl/fl^*. (**A**) Western blots show expression of β-arrestin1 and β-arrestin2 in HUVECs and LECs. (**B**) Schematic images show the generation of *Arrb1/2^ΔiLEC^* embryos by timed mating of *Arrb1/2^fl/fl^* female mice and *Arrb1/2^fl/fl^* *Prox1CreER^T2^* male mice. (**C**) Immunostaining of jugular lymphatic sacs (JLSs) shows decreased β-arrestin1 (yellow arrowheads) and β-arrestin2 (white arrows) expression in the *Arrb1/2^ΔiLEC^* and *Arrb1/2^fl/fl^* embryos at E15.5. Scale bar: 20 μm. (**D**) Representative images of *Arrb1/2^fl/fl^* and *Arrb1/2^ΔiLEC^* embryos at E15.5. Yellow asterisks indicate edema areas; white arrowheads indicate hemorrhagic spots. Scale bar: 5 mm. (**E**) Quantification of edema index normalized to crown rump length (CRL); *n* = 16–20 embryos per group. Gray circles represent *Arrb1/2^fl/fl^* embryos, white squares represent edematous *Arrb1/2^ΔiLEC^* embryos, and gray squares represent non-edematous *Arrb1/2^ΔiLEC^* embryos. Unpaired 2-tailed Student’s *t* test. *P* values are indicated on the graphs. (**F**) H&E staining shows enlarged and blood-filled JLS of *Arrb1/2^ΔiLEC^* embryo at E15.5. CA, carotid artery; eJV: external jugular vein; Scale bar: 100 μm.

β-Arrestins are ubiquitously expressed throughout the body, including within human dermal LECs, which interestingly exhibited substantially higher levels of protein expression compared with blood endothelial cells (Figure 1A). In addition, single-cell RNA-Seq data of murine lymphatics confirmed expression of β-arrestins in both initial and collecting lymphatics (40). Therefore, to test the role of β-arrestins in embryonic lymphatic development, we generated double β-arrestin1 and β-arrestin2 KO mice using an inducible lymphatic-Cre driver. Mice homozygous for floxed alleles of *Arrb1/β-arrestin1* and *Arrb2/β-arrestin2* (14, 15) were crossed to transgenic mice expressing an inducible *CreER^T2^* under the lymphatic *Prox1* promoter (41). To induce embryonic deletion of both *β-arrestin1* and *-2*, pregnant female dams were administered tamoxifen by oral gavage at E10.5 and E12.5, which are critical time points for lymphatic vascular development (Figure 1B). Lymphatic loss of β-arrestin1 and β-arrestin2 protein expression was confirmed by IHC, revealing markedly reduced to undetectable levels in jugular LECs of *Arrb1/2^fl/fl^* *Prox1CreER^T2^* (*Arrb1/2*^ΔiLEC^) embryos compared with control *Arrb1/2^fl/fl^* embryos (Figure 1C).

Gross examination of E15.5 *Arrb1/2*^ΔiLEC^ embryos showed profound hydrops fetalis (Figure 1D, yellow asterisks), with a high volume of edema in the peripheral dorsal region compared with *Arrb1/2^fl/fl^* embryos. Moreover, some *Arrb1/2*^ΔiLEC^ embryos displayed bursts of hemorrhage within the peripheral edematous regions (Figure 1D, white arrows). Quantitative analysis of edema, as normalized to crown rump length (CRL), showed that approximately half of the *Arrb1/2*^ΔiLEC^ embryos (Figure 1E, white squares) exhibited approximately 4 times higher edema indices compared with non-edematous *Arrb1/2*^ΔiLEC^ embryos (Figure 1E, gray squares) and *Arrb1/2^fl/fl^* controls, driving a statistically significant increase in edema index between genotypes. Histological analysis of the peripheral regions substantiated the presence of interstitial edema in *Arrb1/2*^ΔiLEC^ embryos compared with controls (Figure 1F, red asterisks). Transverse sections through the thoracic region identified the typical anatomy of the carotid artery directly adjacent to the exterior jugular vein and juxtaposed to the peripheral jugular lymph sac in embryos of both genotypes. However, in edematous *Arrb1/2*^ΔiLEC^ embryos, the jugular lymphatic sac was markedly enlarged and dilated, indicative of the pathophysiological consequence of edema. In addition, the jugular lymphatic sacs of *Arrb1/2*^ΔiLEC^ embryos with hemorrhage were also enlarged and abnormally filled with red blood cells, likely resulting from the clearance of hemorrhagic interstitial fluid.

**Figure 2:**
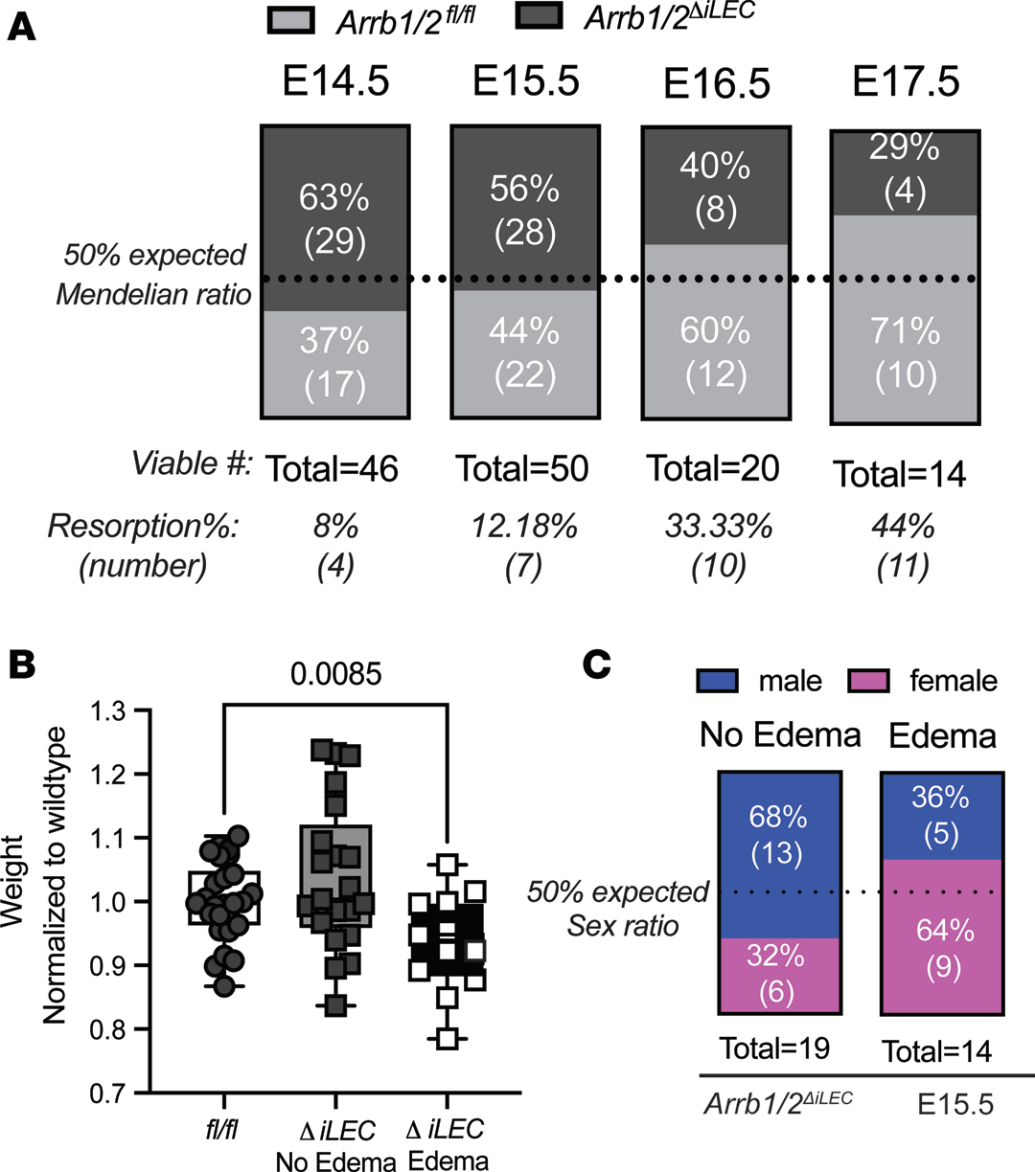
Increased lethality and growth restriction in the *Arrb1/2^ΔiLEC^* embryos compared with *Arrb1/2^fl/fl^*. (**A**) As shown by non-Mendelian ratio, increased resorption embryo number and decreased viable *Arrb1/2^ΔiLEC^* embryos between E14.5 and E17.5 compared with *Arrb1/2^fl/fl^*. (**B**) Quantification of weight of *Arrb1/2^fl/fl^*, non-edematous *Arrb1/2^ΔiLEC^*, and edematous *Arrb1/2^ΔiLEC^* embryos at E15.5. Weight of embryos was normalized to the *Arrb1/2^fl/fl^* embryos from same litter; *n* = 12–50 embryos per group. One-way ANOVA. *P* value is indicated on the graph. (**C**) Sex ratio in non-edematous and edematous *Arrb1/2^ΔiLEC^* embryos at E15.5.

Interestingly, the embryonic hydrops fetalis phenotype was no longer observed at E16.5 or later gestational time points, suggesting either resolution of edema or demise of affected embryos. Genotyping of embryos from E14.5 to E17.5 showed a precipitous decline in the number of *Arrb1/2*^ΔiLEC^ embryos compared with *Arrb1/2^fl/fl^* embryos, skewing away from the expected 50% Mendelian ratio between genotypes (Figure 2A). Indeed, the loss of *Arrb1/2*^ΔiLEC^ embryos was also accompanied by an increase in the total number of resorbed embryos during this gestational time period (Figure 2A), indicating that lymphatic-specific loss of *β-arrestin1* and *β-arrestin2* causes increased midgestational mortality. In support of gestational demise, the weight of edematous *Arrb1/2*^ΔiLEC^ embryos was significantly decreased compared with *Arrb1/2^fl/fl^* embryos at E15.5, further indicative of growth arrest and demise due to lymphatic β-arrestin1/2 deficiency (Figure 2B).

Primary lymphedema is sex-dependent and is 3 times more prevalent in women compared with men (42, 43). To determine whether the hydrops fetalis in the *Arrb1/2*^ΔiLEC^ embryos exhibited sex-dependent penetrance, we performed SRY sex chromosome genotyping. Interestingly, at E15.5, in the non-edematous *Arrb1/2*^ΔiLEC^ embryos, 68% of embryos were male and 32% were female; in the edematous embryos, 36% of embryos were male and 64% were female (Figure 2C), indicating a sex-dependent prevalence of hydrops fetalis caused by lymphatic β-arrestin1/2 deficiency.

Although *Prox1CreER^T2^* mice are a widely used research tool for Cre-mediated excision of floxed genes in lymphatics, cardiomyocytes also express *Prox1* (44). Therefore, we evaluated the anatomy of the embryonic heart at day E15.5 by H&E and found no differences in heart size or structure between the *Arrb1/*2^ΔiLEC^ and control embryos (Supplemental Figure 1; supplemental material available online with this article; https://doi.org/10.1172/jci.insight.198032DS1). Cardiac atria, ventricles, valves, and septum appeared normal in all animals evaluated. We also found no evidence of pericardial effusion, even among *Arrb1/*2^ΔiLEC^ embryos with edema. Collectively, these data support the conclusion that lymphatic loss of *β-arrestin1* and *-2* causes increased incidence of edematous hydrops fetalis with occasional interstitial hemorrhage, which is associated with midgestational growth arrest and embryonic demise.

### Loss of β-arrestin1 and -2 decreases lymphatic proliferation in vivo and in vitro through distinct signaling pathways.

**Figure 3:**
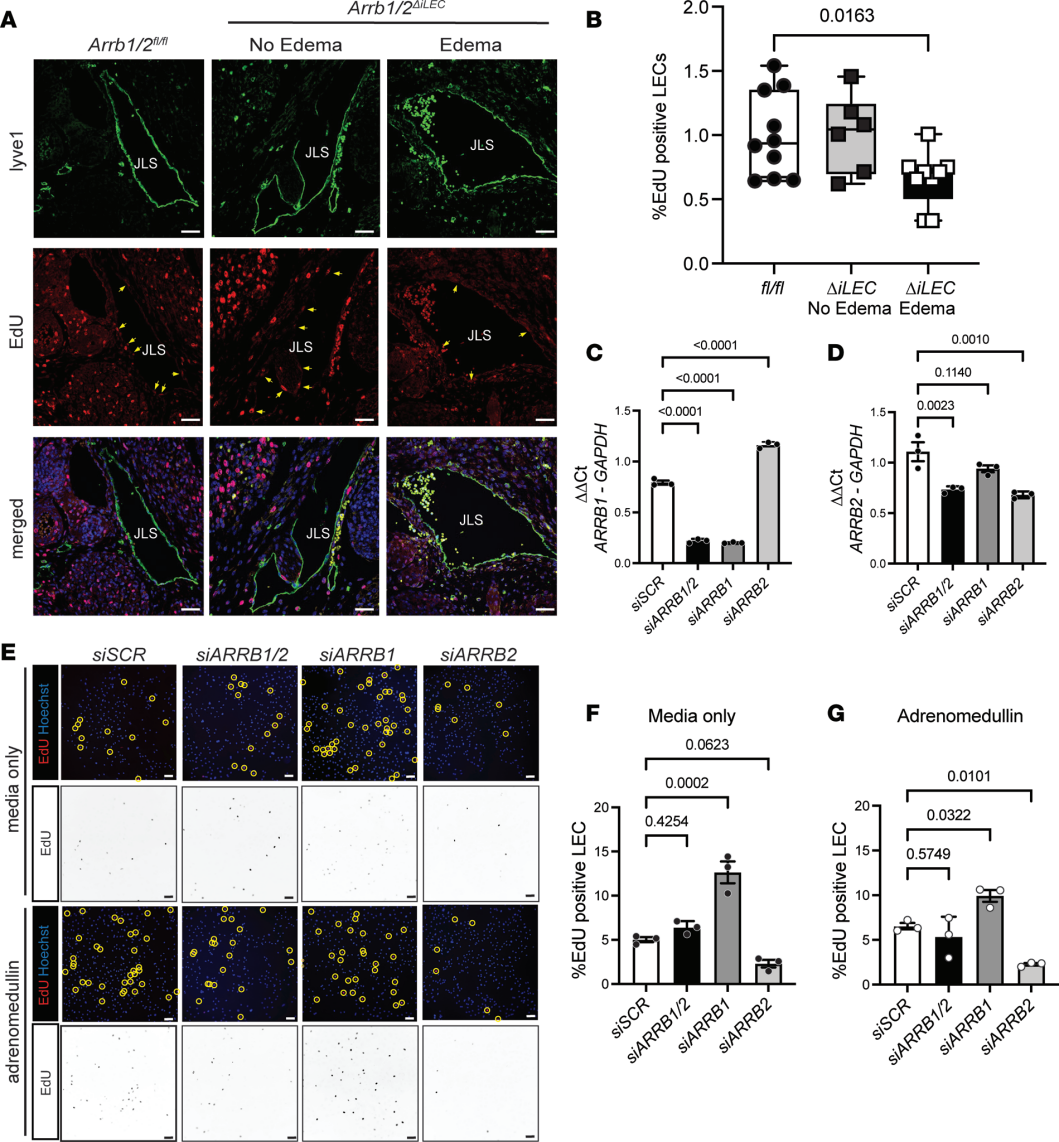
Lymphatic loss of *Arrb1/2* causes decreased proliferation of the jugular lymphatic vessels. (**A** and **B**) Representative images (**A**) and quantification (**B**) of EdU proliferation assay and immunostaining in the JLS sections of *Arrb1/2^fl/fl^* and *Arrb1/2^ΔiLEC^* embryos at E15.5. Yellow arrows: EdU-positive LECs. *n* = 6–10 embryos per group. One-way ANOVA. (**C** and **D**) Quantification of *ARRB1* (**C**) and *ARRB2* (**D**) mRNA expression in LECs transfected with scramble, *ARRB1*, *ARRB2*, or *ARRB1/2* siRNAs. (**E**–**G**) Representative images (**E**) and quantification (**G**) of EdU-positive proliferation of LECs transfected with scramble siRNA (siSCR), *ARRB1/2*, *ARRB1*, *ARRB2*, and siRNAs and stimulated without (**F**) or with adrenomedullin (**G**). Scale bar: 20 μm. One-way ANOVA. *P* values are indicated on the graphs.

To determine whether double KO of β-arrestin1 and β-arrestin2 affected embryonic LEC proliferation, we utilized an EdU proliferation assay. Briefly, EdU was administered by oral gavage to pregnant dams, and embryos were dissected 4 hours later, followed by fixation, sectioning, and quantification of EdU incorporation. At E15.5, edematous *Arrb1/2*^ΔiLEC^ embryos exhibited a significant decrease in the number of EdU^+^Lyve1^+^ LECs in the jugular lymphatic sac compared with *Arrb1/2^fl/fl^* controls and non-edematous *Arrb1/2*^ΔiLEC^ embryos (Figure 3, A and B). These results indicate that loss of β-arrestin1 and -2 decreases LEC proliferation in vivo within edematous mice.

To delineate the individual and/or combined effects of β-arrestin1 and β-arrestin2 on LEC proliferation, siRNA transfection in human dermal LECs was utilized to efficiently knockdown β-arrestin1 (si*ARRB1*), β-arrestin2 (si*ARRB2*), or both (si*ARRB1/2*) in combination (Figure 3, C and D). EdU incorporation was then measured in cells grown under media-only conditions or under a proliferative stimulus. LEC proliferation was stimulated with AM, a mitogenic peptide hormone that promotes LEC proliferation through the GPCR *CALCRL* and CLR (protein), via ERK/CREB pathways (45, 46) (Supplemental Figure 2A). Interestingly, knockdown of β-arrestin1 significantly increased the number of EdU-positive cells grown under media conditions and under AM stimulation compared with control cells (siScramble, siSCR) (Figure 3, E–G). Knockdown of β-arrestin2 or double knockdown of β-arrestin1/2 (*siARRB1/2*) did not show significant differences in EdU incorporation (Figure 3, E and F) compared to control LECs under media conditions. However, knockdown of β-arrestin2 in LECs resulted in a significant decrease in LEC proliferation after AM stimulation (Figure 3, E and G). Together, the opposing effects of β-arrestin1 versus -2 knockdown on LEC proliferation imply differential downstream signaling effects.

**Figure 4:**
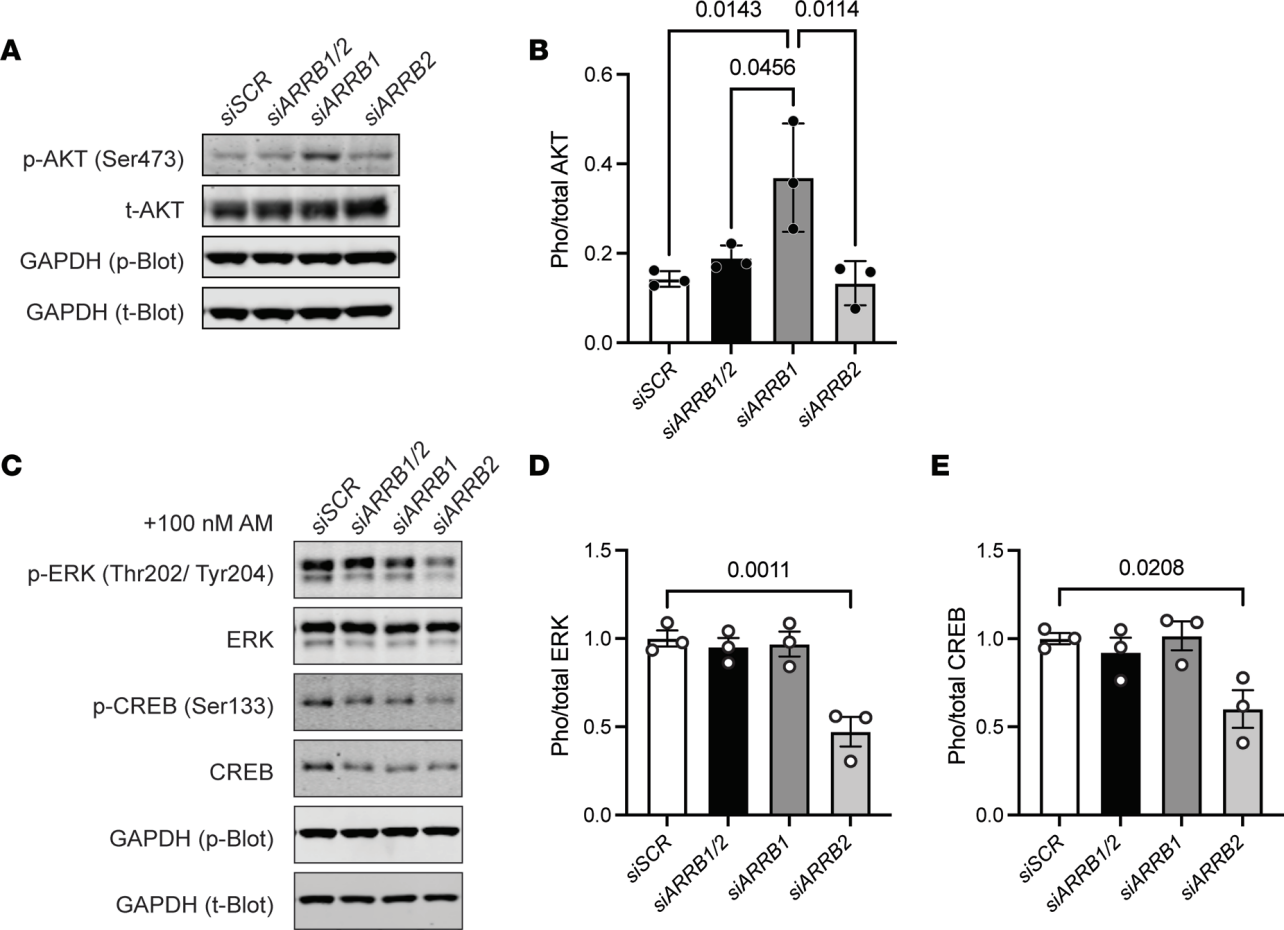
Signaling changes in LECs caused by deficiency of combined or individual β-arrestins. (**A**) Representative Western blot images showing AKT phosphorylation at Ser473 and total AKT levels in LECs transfected with scramble control, ARRB1, ARRB2, or ARRB1/2 siRNAs. (**B**) Quantification of p-AKT to total AKT in LECs transfected with scramble, *ARRB1*, *ARRB2*, or *ARRB1/2* siRNAs. Each dot represents 1 biological replicate. One-way ANOVA. (**C**) Representative Western blot images show phosphorylation of ERK1/2 at Thr202/Tyr204, total ERK, phosphorylation of CREB at Ser133, total CREB level in LECs transfected with scramble, *ARRB1*, *ARRB2*, or *ARRB1/2* siRNAs after AM stimulation. (**D** and **E**) Quantification of p-ERK to total ERK, p-CREB to total CREB in LECs transfected with scramble, *ARRB1*, *ARRB2*, or *ARRB1/2* siRNAs. Each dot represents 1 biological replicate. One-way ANOVA. *P* values are indicated on the graphs.

In support of this notion, downstream signaling assays showed that knockdown of β-arrestin1 alone resulted in a significant increase in AKT phosphorylation at Ser473 under media conditions compared with control cells (siSCR) (Figure 4, A and B), which correlates with the increased proliferation observed upon loss of β-arrestin1 (Figure 3, E and F). In contrast, knockdown of β-arrestin1 or double knockdown of β-arrestin1/2 did not significantly alter AM-induced ERK or CREB phosphorylation compared with similarly treated control cells (siSCR). Consistent with the documented role of p-ERK and p-CREB in governing AM-mediated LEC proliferation, knockdown of β-arrestin2 in LECs resulted in a significant decrease in AM-induced phosphorylation of ERK (Thr202/Tyr204) and CREB (Ser133) compared with similarly treated control cells (siSCR) (Figure 4, C–E). Taken together, these data support essential functions for both β-arrestin1 and -2 in governing LEC proliferation, each exploiting different downstream signaling pathways under basal or mitogen-induced conditions.

### Loss of β-arrestin1 and -2 alters expression of some lymphatic GPCRs in vitro.

Depending on ligand bias and the relative affinity of β-arrestin1 or -2 to individual GPCRs, β-arrestins can influence receptor expression. To delineate the individual and/or combined effects of β-arrestin1 and β-arrestin2 knockdown on GPCR expression in human dermal LECs, we transfected LECs with siRNAs to knock down β-arrestin1 (*siARRB1*), β-arrestin2 (*siARRB2*), or both (*siARRB1/2*) in combination (Figure 3, C and D). Then, the expression of several GPCRs, including CALCRL, ACKR3, APLNR, and GPRC5B, was determined by qRT-PCR. Interestingly, the mRNA expression of ACKR3 and GPRC5B was decreased by knockdown of β-arrestin1 and increased by knockdown of β-arrestin2, whereas no changes were found in the LECs with combined β-arrestin1 and -2 knockdown (Supplemental Figure 3, A and B). Conversely, the mRNA expression of CALCRL and APLNR was not changed by single nor combined knockdown of β-arrestin1 and/or -2 (Supplemental Figure 3, C and D). These results indicate district roles of β-arrestin1 and -2 in regulating the expression of GPCRs in LECs, potentially reflecting differential preferences for G-protein–biased or β-arrestin–biased signaling.

### Edematous Arrb1/2^ΔiLEC^ embryos exhibit dilated lymphatic vessels and dysregulated VE-cadherin adherens junction morphology.

**Figure 5:**
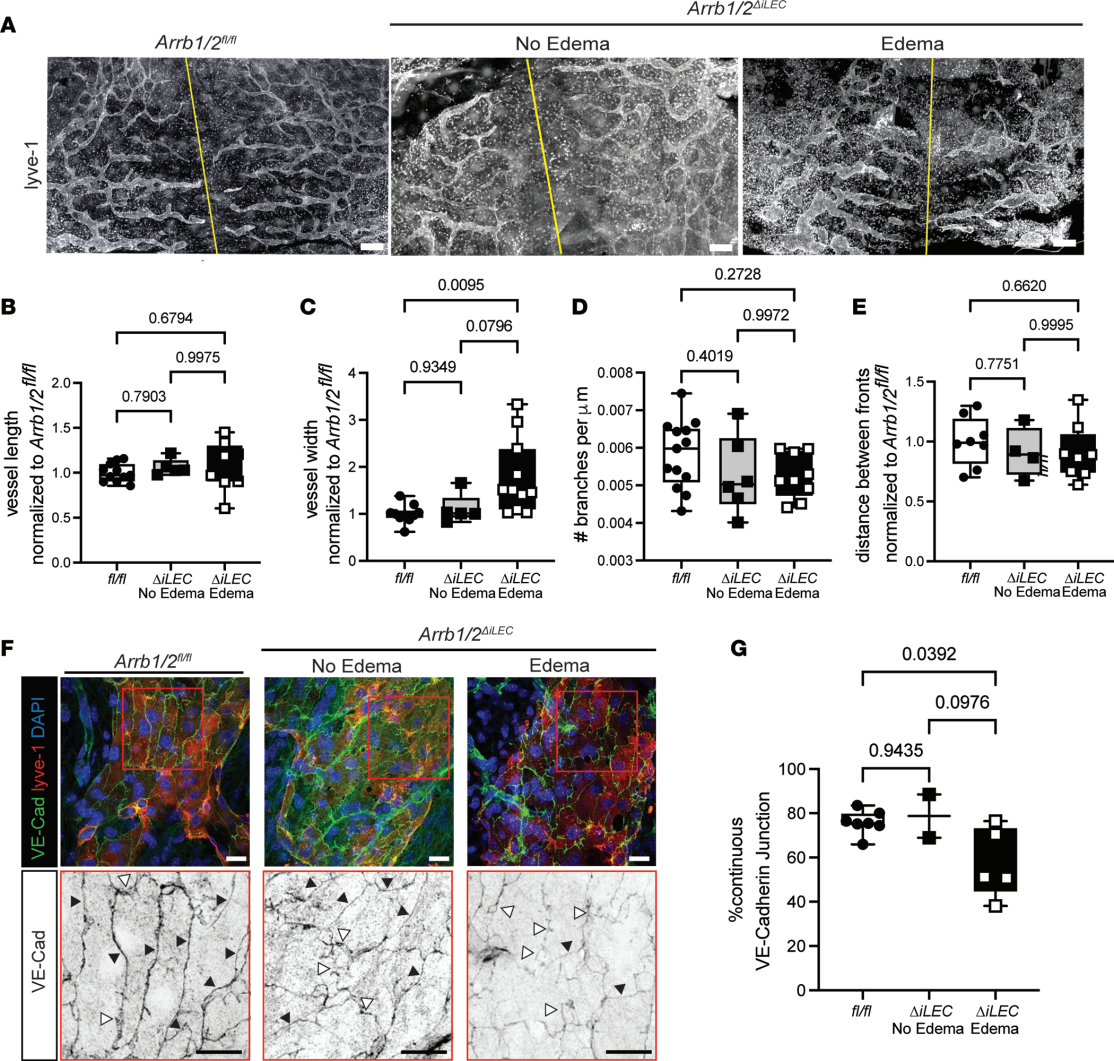
Dorsal dermal lymphatic vessels are dilated in *Arrb1/2^ΔiLEC^* embryos. (**A**) Representative images of whole-mount immunostaining with Lyve-1 antibody of dorsal skin from *Arrb1/2^fl/fl^*, non-edematous *Arrb1/2^ΔiLEC^*, and edematous *Arrb1/2^ΔiLEC^* embryos at E15.5. Yellow line indicates dorsal midline. Scale bar: 100 μm. (**B**–**E**) The width (**C**) of the lymphatic vessel in the edematous *Arrb1/2^ΔiLEC^* embryos was increased compared with control *Arrb1/2^fl/fl^* embryos at E15.5. No differences were observed in the vessel length (**B**), branches (**D**), and distance between fronts (**E**) between 3 groups. *n* = 6–11 embryos per group. One-way ANOVA. (**F** and **G**) Representative images (**F**) and continuous VE-cadherin junction quantification (**G**) of whole-mount immunostaining with VE-cadherin and Lyve-1 antibodies of lymphatic vessel in embryonic dorsal skin. Black arrows, continuous junctions; open arrows, discontinuous junctions. Scale bar: 20 μm. *n* = 2–7 embryos per group. One-way ANOVA. *P* values are indicated on the graphs.

During lymphatic expansion, proximal dermal vessels migrate toward the dorsal midline. Whole-mount staining of the dorsal skin with the lymphatic marker Lyve-1 revealed a complex meshwork of lymphangiogenesis extending toward the midline in both control *Arrb1/2^fl/fl^* and *Arrb1/2*^ΔiLEC^ embryos, regardless of the presence of edema (Figure 5A). Quantification confirmed no significant differences in the vessel length, number of branches, or distance between vessel migrating fronts between edematous *Arrb1/2*^ΔiLEC^, non-edematous *Arrb1/2*^ΔiLEC^, and *Arrb1/2^fl/fl^* control embryos (Figure 5, A, B, D, and E). However, edematous *Arrb1/2*^ΔiLEC^ embryos showed statistically significant increases in lymphatic vessel width compared with control *Arrb1/2^fl/fl^* embryos and non-edematous *Arrb1/2*^ΔiLEC^ embryos (Figure 5, A and C). Thus, dilation of the lymphatic vasculature in edematous *Arrb1/2*^ΔiLEC^ is consistent with the pathological presence of edema and suggests that lymphatic loss of β-arrestin1/2 functionally impairs the ability of lymphatics to maintain fluid homeostasis.

VE-cadherin is the principal adherens junction protein of lymphatics, regulating endothelial barrier function and permeability. Previous studies have shown that β-arrestins directly interact with VE-cadherin to regulate its internalization and increase vessel permeability (18–20). Thus, loss of β-arrestins in LECs could destabilize VE-cadherin junctions, thereby disrupting lymphatic vessel permeability and function. To test this hypothesis, we co-stained the embryonic dorsal skin with both VE-cadherin and Lyve1 and scored the number of continuous (black arrows) and noncontinuous (open arrows) VE-cadherin cell-cell junctions (Figure 5F). Consistent with the pathological findings, we found that edematous *Arrb1/2*^ΔiLEC^ embryos displayed a significant reduction in continuous, zipper-like VE-cadherin adherens junctions compared with control *Arrb1/2^fl/fl^* and non-edematous *Arrb1/2*^ΔiLEC^ embryos (Figure 5, F and G).

### β-Arrestin2 primarily drives VE-cadherin membrane assembly under basal conditions in vitro.

**Figure 6:**
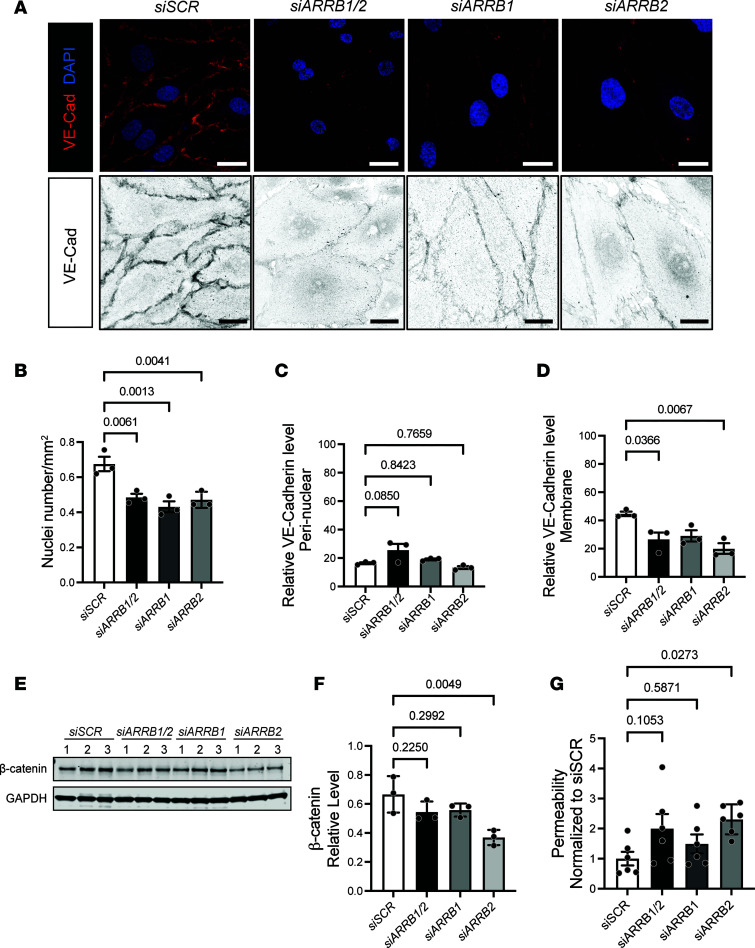
Diminished VE-cadherin adherens junctions in the membrane of LECs cause loss of β-arrestin1 or β-arrestin2. (**A**) Representative images of VE-cadherin antibody staining in LECs transfected with scramble siRNA (siSCR), *ARRB1/2*, *ARRB1*, and *ARRB2* siRNA. Scale bar: 20 μm. (**B**) Nuclei number per square millimeter of LECs transfected with scramble siRNA (siSCR), *ARRB1/2*, *ARRB1*, and *ARRB2* siRNA. *n* = 3 biological replicates per group. One-way ANOVA. (**C** and **D**) Quantification of perinuclear (**C**) and membrane (**D**) localized VE-cadherin intensity in LECs transfected with scramble siRNA (siSCR), *ARRB1/2*, *ARRB1*, and *ARRB2* siRNA. *n* = 3 biological replicates per condition. One-way ANOVA. *P* ≤ 0.05 compared with siSCR. (**E** and **F**) Western blot images (**E**) and quantification (to GAPDH) show β-catenin protein levels in cell membranes enriched from LECs transfected with scramble, *ARRB1*, *ARRB2*, or *ARRB1/2* siRNAs. *n* = 3 biological replicates per group. One-way ANOVA. (**G**) Relative permeability coefficient of 2,000 kDa FITC-dextran of LECs transfected with scramble siRNA (siSCR), *ARRB1/2*, *ARRB1*, and *ARRB2* siRNA. *n* = 6 biological replicates per group. One-way ANOVA. *P* values are indicated on the graphs.

To further distinguish the roles of β-arrestin1 and β-arrestin2 in regulating VE-cadherin adherens junctions, we knocked down β-arrestin1/2 by siRNA in cultured primary human LECs and evaluated the structure and function of cell-cell junctions. First, we noted a statistically significant increase in cell size with single and double β-arrestin knockdown in LECs compared with LECs transfected with siSCR, indicated by decreased cell nuclei numbers per square millimeter (Figure 6, A and B). Next, we noted that under basal growth conditions, VE-cadherin staining intensity at cellular junctions was markedly decreased in single and double β-arrestin1/2 knockdown cells compared with siSCR-treated cells (Figure 6A). Although knockdown of β-arrestin1/2 did not change total VE-cadherin protein levels (Supplemental Figure 4), we identified changes in its subcellular localization. Specifically, image intensity measurements of VE-cadherin showed no significant changes in perinuclear levels across all conditions (Figure 6C) but statistically significant reductions in membrane VE-cadherin levels with loss of β-arrestin2 and a combination of β-arrestin1/2 (Figure 6D). To further confirm these results, membrane proteins were enriched from control LECs (siSCR) and LECs with knockdown of β-arrestin1 (siARRB1) and/or β-arrestin2 (siARRB2) and probed for membrane expression of β-catenin, a direct membrane VE-cadherin protein interactor. We found that LECs with β-arrestin2 knockdown exhibited a significant reduction in membrane β-catenin levels compared with siSCR-treated controls (Figure 6, E and F).

To determine functional changes due to β-arrestin1/2 deficiency in LECs, we assessed the permeability of LECs transfected with control siRNA (siSCR), β-arrestin1 (*siARRB1*), and/or β-arrestin2 (*siARRB2*) and treated with 2,000 kDa FITC-dextran. Knockdown of β-arrestin2 (*siARRB2*) significantly increased LEC permeability compared with siSCR-treated LECs (Figure 6G). Taken together, these data support that under basal conditions in vitro, β-arrestin1/2 regulate LEC cell size, VE-cadherin membrane expression and subcellular localization, and LEC permeability, and that this phenotype is primarily driven by β-arrestin2.

### β-Arrestins are required for GPCR-mediated organization of VE-cadherin into continuous junctions in vitro.

**Figure 7:**
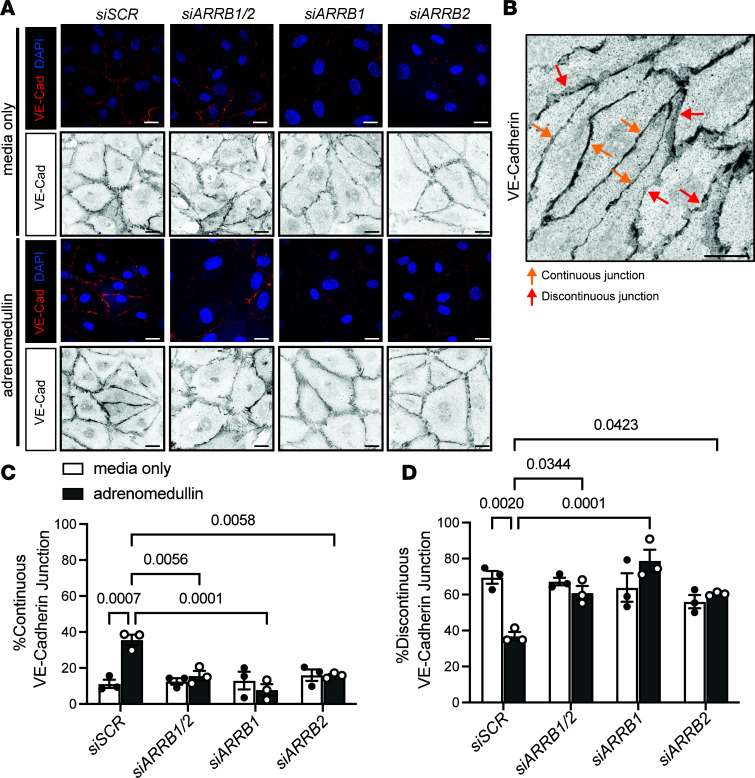
β-Arrestins are required for GPCR-mediated organization of VE-cadherin into continuous junctions in vitro. (**A**) Representative images of VE-cadherin antibody staining in LECs transfected with scramble siRNA (siSCR), *ARRB1/2*, *ARRB1*, and *ARRB2* siRNA and cultured in media only or stimulated with adrenomedullin. Scale bar: 20 μm. (**B**) Representative image to show the criteria of the continuous and discontinuous VE-cadherin adherens junctions. Yellow arrows, continuous junctions; red arrows, discontinuous junctions. Scale bar: 20 μm. (**C** and **D**) Quantification of the percentage of continuous (**C**) and discontinuous (**D**) VE-cadherin adherens junctions in LECs transfected with scramble siRNA (siSCR), *ARRB1/2*, *ARRB1*, and *ARRB2* siRNA and cultured in media only or stimulated with adrenomedullin. *n* = 3 biological samples per group. Two-way ANOVA. *P* values are indicated on the graphs.

We have previously shown that activation of the GPCR CALCRL by its ligand AM rapidly and potently promotes VE-cadherin adherens junction remodeling by converting discontinuous, button-like junctions into continuous, zipper-like junctions (47–49). To determine whether β-arrestins are required for GPCR-mediated reorganization of VE-cadherin adherens junctions in LECs, we used AM as a tool compound. As expected, treatment of control siSCR cells with AM resulted in linearization of VE-cadherin junctions into a continuous, zipper-like morphology compared with media-only siSCR controls (Figure 7A). Conversely, LECs with knockdown of β-arrestin1/2 (*siARRB1/2*), β-arrestin1 (*siARRB1*), and β-arrestin2 (*siARRB2*) exhibited reduced levels of VE-cadherin membrane expression and prevented AM-induced VE-cadherin adherens junction linearization (Figure 7, A–C), with statistically significant reductions in the percentage of continuous junctions and concomitant significant increases in discontinuous junctions compared with AM-treated siSCR control cells (Figure 7, C and D). Collectively, these data demonstrate that β-arrestins are required for reorganization of VE-cadherin adherens junctions after GPCR stimulation.

## Discussion

**Figure 8:**
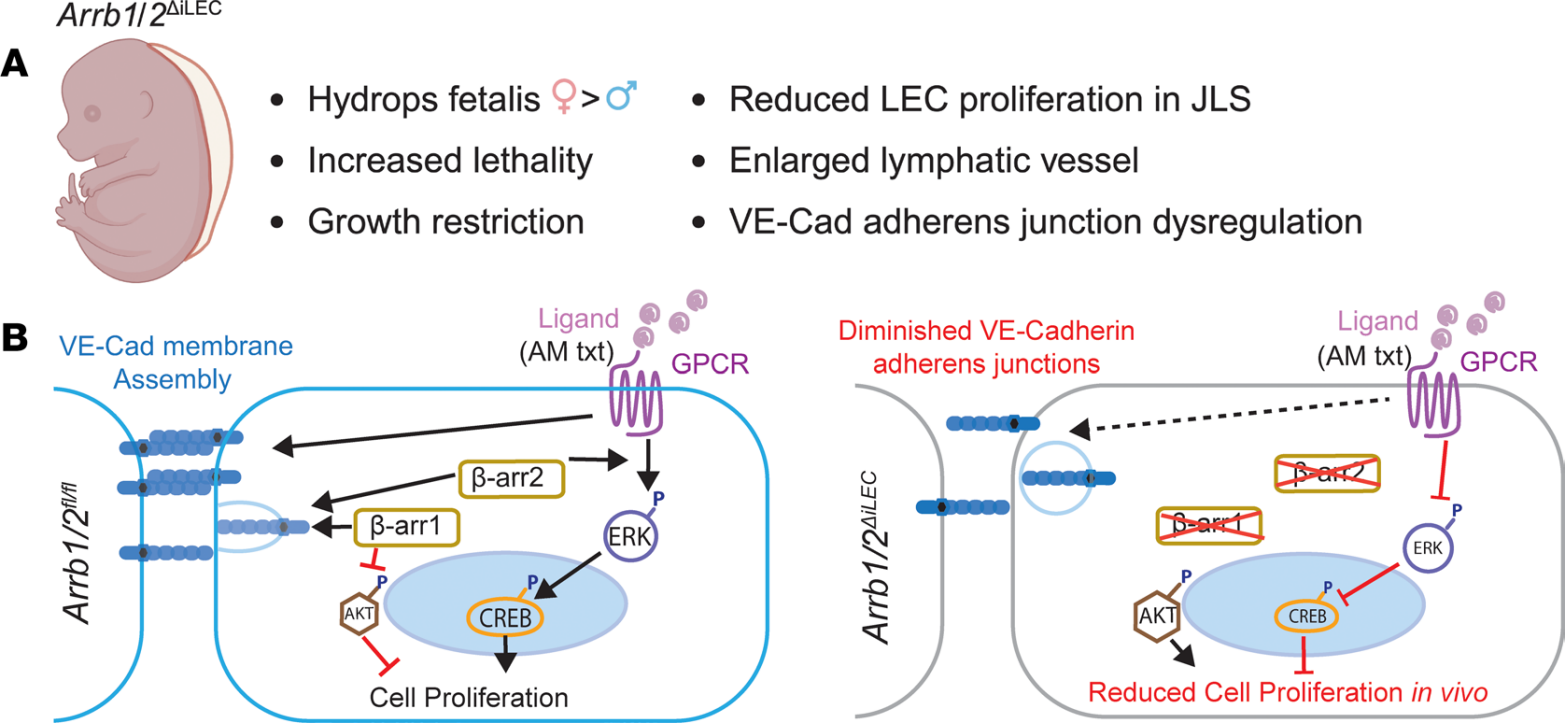
Schematic outlining how β-arrestin1 and β-arrestin2 differentially regulate LEC proliferation and VE-cadherin adherens junctional remodeling. (**A**) *Arrb1/2^ΔiLEC^* embryos display hydrops fetalis, increased lethality, and growth restriction compared with *Arrb1/2^fl/fl^* controls. This is accompanied by reduced LEC proliferation within the jugular lymphatic sac, dilated lymphatic vessels, and dysregulated VE-cadherin adherens junction assembly at E15.5. Created with BioRender.com, released under a Creative Commons Attribution-NonCommercial-NoDerivatives 4.0 International license. (**B**) In human dermal LECs, β-arrestin1 deficiency enhances cell proliferation, potentially through the phosphorylation of AKT at Ser473. Conversely, β-arrestin2 deficiency decreases AM-induced proliferation, potentially due to disruption of AM-mediated ERK and CREB activation. Finally, β-arrestin1/2 are essential for VE-cadherin localization to LEC adherens junctions, whereby β-arrestin2 and β-arrestin1/2 deficiency decreases VE-cadherin membrane localization, and β-arrestin2 deficiency increases LEC permeability. Deficiency of single or double β-arrestin also reduces AM-induced VE-cadherin junction linearization. Black lines with arrow, increased effect; red lines with blunt head, decreased effect; dashed line, prevented effect.

This study identifies a critical role for β-arrestin1 and β-arrestin2 in lymphatic vessel development and defines an essential role for lymphatic β-arrestin1/2 in embryonic development and survival. Specifically, we found in vivo that loss of β-arrestin1/2 resulted in midgestational hydrops fetalis, growth restriction, and increased embryonic mortality of *Arrb1/2*^ΔiLEC^ embryos (Figure 8). The observation that a subset of the *Arrb1/2*^ΔiLEC^ embryos developed edema may be due to variable KO efficiency of β-arrestin1 and β-arrestin2 between individual embryos.

To define the mechanisms by which β-arrestin1 and β-arrestin2 regulate lymphatic signaling, we employed human LECs with β-arrestin1 and/or β-arrestin2 knockdown. We found in vitro that β-arrestin1 deficiency resulted in increased proliferation, increased phosphorylation of AKT, and decreased VE-cadherin membrane localization. In contrast, we found that β-arrestin2 deficiency decreased AM-induced proliferation, decreased AM-induced phosphorylation of ERK and CREB, decreased VE-cadherin membrane localization, and increased LEC permeability. Consistent with these findings, AM failed to linearize cell-cell VE-cadherin adherens junctions in LECs with single or double β-arrestin knockdown (Figure 8). Taken together, these results demonstrate that β-arrestin1 and β-arrestin2 engage distinct signaling modalities to regulate lymphatic function.

*Arrb1/2*^ΔiLEC^ embryos with edema displayed an enlarged jugular lymphatic sac and dilated dorsal skin lymphatics compared with those of *Arrb1/2^fl/fl^* embryos at E15.5. However, edematous *Arrb1/2*^ΔiLEC^ embryos also exhibited decreased cellular proliferation compared with *Arrb1/2^fl/fl^* embryos. These phenotypes may be reconciled due to compensatory cell hypertrophy, which was also evident in the increased cell size of cultured cells with knockdown of β-arrestin1/2 compared with siRNA-treated cells. Another possibility is that the dilated lymphatic vessels arise from defective endothelial cell rearrangement caused by disrupted VE cadherin–mediated adherens junction assembly or remodeling, which is essential for maintaining lymphatic barrier integrity and regulating vessel permeability.

Loss of β-arrestin1/2 resulted in lymphedema in a sex-dependent manner: 64% of edematous *Arrb1/2*^ΔiLEC^ embryos at E15.5 were female and only 36% were male. Sex-specific gonadal differentiation occurs around E12.5 in the mouse, with male-specific testosterone production commencing between E12 and E13, and female ovarian steroidogenesis delayed until P7. Thus, the underlying hormonal and/or chromosomal mechanisms leading to the observed sex differences in *Arrb1/2*^ΔiLEC^ embryos may be complex. It has long been demonstrated that hereditary primary lymphedema is 3 times more likely to occur in women compared with men (50, 51). For example, Milroy disease, a form of primary lymphedema caused by VEGFR-3 mutations, has a higher prevalence in women compared with men (52). Furthermore, Turner syndrome, another form of primary lymphedema, is the most common sex chromosome abnormality affecting women (53, 54). Future studies will unravel the contributions of development, hormones, and sex chromosomes to the pathology of lymphatic-associated diseases.

β-Arrestin1/2 regulate cellular proliferation in multiple cellular contexts. For example, recruitment of β-arrestin to nicotinic acetylcholine receptors is essential for nicotine-induced cell proliferation (55). After carotid injury, increased ERK activation and medial smooth muscle cell proliferation have been observed in β-arrestin1 KO mice, and proliferation was decreased in β-arrestin2 KO mice (56). Conversely, in patients with gastric cancer, overexpression of β-arrestin1 is associated with poor prognosis, and its knockdown decreases tumor cell proliferation. Therefore, given the multifunctionality of β-arrestin1/2, it is not surprising that the roles of β-arrestins are complex and context dependent.

In the current study, double KO of β-arrestin1 and β-arrestin2 in LECs decreased proliferation in the jugular lymphatic sac at E15.5. However, although no significant difference in proliferation was observed in the serum-starved or AM-stimulated human dermal LECs after double knockdown of β-arrestin1 and -2, there was a differential effect on proliferation and signaling with knockdown of either β-arrestin1 or -2 alone. Specifically, knockdown of β-arrestin1 in LECs in vitro increased cell proliferation, which coincided with enhanced phosphorylation of AKT at Ser473. These data are aligned with previous reports that have linked β-arrestin1 to AKT signaling, for example, in ghrelin-induced AKT phosphorylation at Ser473 (57) and in G protein β1γ2 subunit interactions to promote AKT signaling and proliferation (58). Conversely, in the context of mouse embryonic fibroblasts, both β-arrestin1 and β-arrestin2 have been reported to inhibit AKT signaling by facilitating PTEN lipid phosphatase activity (59), which further supports that β-arrestin1 and -2 have both cell- and context-dependent functions. Finally, GPCR–β-arrestin scaffolding promotes cytosolic ERK1/2 activation, leading to ERK1/2 nuclear translocation and interaction with nuclear substrates such as CREB to drive proliferation (60–62). In line with these results, we found that knockdown of β-arrestin2, but not β-arrestin1, reduced AM-induced phosphorylation of ERK (Thr202/Tyr204) and CREB (Ser133), suggesting that β-arrestin2 specifically regulates AM-induced MAPK signaling cascades in LECs.

β-Arrestins regulate VE-cadherin adherens junctions via multiple mechanisms (Figure 8). First, β-arrestin2 can directly interact with VE-cadherin at residues Y685 and S665 to modulate its internalization and affect vessel permeability (18–20). Second, β-arrestins participate in VEGFR3-mediated disruption of endothelial barrier function through promoting rapid VE-cadherin endocytosis in blood endothelial cells (17, 18). Third, in blood endothelial cells, β-arrestins interact with activated GPCRs, such as the β2 adrenergic receptor, to promote recruitment of VE-cadherin and β-catenin to the endothelial cell membranes, thereby increasing vascular permeability (17). Fourth, we have previously shown that AM rapidly linearizes VE-cadherin adherens junctions by converting highly permeable, button-like junctions into impermeable, zipper-like junctions. This process depends on β-arrestin recruitment to AM-stimulated CALCRL to promote cellular internalization of the ligand-activated complex (63, 64).

Notably, during lymphangiogenesis between E10.5 and E15.5, the VE-cadherin adherens junctional conformation is exclusively continuous and later transitions to discontinuous button-like junctions around E16.5 in the dermal initial lymphatics to facilitate fluid uptake (25). To this end, we found that edematous *Arrb1/2*^ΔiLEC^ embryos exhibited a decrease in continuous, zipper-like junctions compared with the *Arrb1/2^fl/fl^* controls at E15.5, along with changes in cell shape. These data suggest that lymphatic loss of β-arrestin1/2 increases embryonic fluid accumulation due to dysregulation of VE-cadherin adherens junctions. These results were partially recapitulated in vitro, whereby knockdown of β-arrestin1/2 in human LECs disrupted VE-cadherin membrane assembly. Specifically, we found that knockdown of β-arrestin2 and double knockdown of β-arrestin1/2 decreased VE-cadherin membrane localization, and that knockdown of β-arrestin1/2 prevented AM-induced linearization of VE-cadherin junctions and increased LEC size. Finally, we found that knockdown of β-arrestin2 increased cell permeability. Taken together, these findings highlight the essential and multifaceted functions of β-arrestin1 and 2 in controlling VE-cadherin dynamics in LECs, ultimately influencing cellular signaling and physiological outcomes.

## Methods

### Sex as a biological variable.

In vivo experiments were conducted on both male and female embryos. To determine the sex of embryos, we performed SRY (Y chromosome) genotyping on archived DNA samples.

### Animals.

To generate *Arrb1* and *Arrb2* double KO mice specifically in lymphatics, the *Arrb1^fl/fl^* and *Arrb2^fl/fl^* (*Arrb1/2^fl/fl^*) mouse was obtained from Sudar Rajogopal (Duke University, Durham, North Carolina, USA) (17), and the *Prox1CreER^T2^* mouse was provided by Taija Makinen (Meilahti Academic Medical Center, Helsinki, Finland) (41). Prior to timed mating, 4- to 6-month-old *Arrb1/2^fl/fl^* mice and *Arrb1/2^fl/fl^* *Prox1CreER^T2^* mice were multihoused in a temperature-controlled room with a 12-hour light/12-hour dark cycle and provided ad libitum access to water and standard chow. The *Arrb1/2^fl/fl^* female mice and *Arrb1/2^fl/fl^* *Prox1CreER^T2^* male mice were used for timed mating, with the presence of a copulation plug identifying gestation day 0.5. After mating was detected, pregnant female mice were singly housed. To induce Prox1-Cre expression, pregnant mice were administered 6 μL/g of 20 mg/mL tamoxifen by oral gavage on the morning of E10.5 and E12.5. Embryos were dissected at E14.5, E15.5, E16.5, and E17.5. Both the right and left sides of the embryos were imaged using a Leica MZ16FA stereo microscope. To calculate the edema index, the transparent edema areas in the cervical, pericardial, and dorsal trunk areas, as well as the CRL of both sides of each embryo, were measured by an observer using Fiji under a blinded protocol. Then, the edema areas on each side were summed. For each embryo, we calculated the average of the 2 sides and divided by the CRL. Edema index = edema area / CRL. Data are presented as mean ± SEM. One-way ANOVA was used for statistical analysis. Tails of the embryos were cut for DNA isolation and genotyping. Then, embryos were fixed in 4% paraformaldehyde (PFA) overnight. Predissection, the female mice were weighed and euthanized via CO_2_ asphyxiation with cervical dislocation as a secondary method.

### Histology.

Mouse embryos were fixed in 4% PFA overnight and embedded in paraffin for sectioning. Then, 5 μm paraffin sections of 4 chambers of the heart and jugular lymphatic sacs were obtained for H&E staining. Sectioning and H&E staining were performed by the UNC Histology Research Core Facility. For IHC staining, the 5 μm paraffin sections of jugular lymphatic sacs were first deparaffinized using CitriSolv, 100% ethanol, 95% ethanol, 70% ethanol, and PBS. Then, the sections were permeabilized by PBS with 0.1% Triton X-100 (PBST) and blocked with 5% normal donkey serum (NDS) in 0.1% PBST. Two sections from each embryo were stained with rabbit anti-arrestin1 antibody (Novus Biologicals, NBP2-67510, 1:200) or rabbit anti-arrestin2 antibody (Thermo Fisher Scientific, PA1-732) and goat anti-mouse Lyve1 antibody (R&D Systems, AF2125, 1:200) overnight at 4°C. Sections were washed with PBST 3 times (5 min/time) and incubated with secondary antibodies: Alexa Fluor 594 AffiniPure Donkey Anti-Rabbit IgG (H+L), Jackson ImmunoResearch, catalog 711-585-152; Alexa Fluor 488 AffiniPure Donkey Anti-Goat IgG (H+L), Jackson ImmunoResearch, catalog 705-545-003; 1:400 in blocking solution. After washing with PBST 3 times for 5 min/time, sections were mounted with ProLong Gold Antifade Mounting Media with DAPI (Thermo Fisher Scientific) and imaged using a ZEISS 800 upright confocal microscope (Hooker Imaging Core, UNC Chapel Hill).

### In vivo EdU proliferation assay.

First,100 μg/g body weight of EdU (Thermo Fisher Scientific, Click-iT EdU Alexa Fluor 594 Imaging kit, C10339) was i.p. injected into pregnant mice 4 hours before euthanasia. Then, mice were euthanized via CO_2_ asphyxiation with cervical dislocation as a secondary method. Embryos were dissected, fixed in 4% PFA overnight, and embedded in paraffin for sectioning. Next, 5 μm sections were stained with Lyve 1 antibody (Rabbit anti Lyve1 antibody, Biosynth, catalog 70R-LR005, 1:200) and secondary antibody [Donkey anti-Rabbit IgG (H+L) Cross-Adsorbed Secondary Antibody, DyLight 488, Thermo Fisher Scientific, catalog SA5-10038], and staining of EdU-positive and cell nuclei was performed following the Click-iT EdU Alexa Fluor 594 Imaging kit manual.

### Whole-mount immunostaining.

This step was performed as previously reported, with slight modifications (46). Briefly, mouse embryos at E15.5 were fixed in 4% PFA overnight and rinsed in PBS, and embryo torsos were transferred in 100% methanol and stored at –20°C. Embryonic back skin was dissected and rehydrated by incubation in a graded series of MeOH/PBS-T (PBS + 0.2% Triton X-100) (75%, 50%, 25%) for 5 minutes each. Samples were washed with PBST for 5 minutes and blocked with blocking buffer (10% NDS in PBST) for 2 hours at room temperature, followed by incubation with primary antibodies (rabbit anti-lyve1, Biosynth, 1:200; Goat anti- mouse VE-Cadherin Antibody, R&D Systems, catalog AF1002) overnight at 4°C. Samples were washed in 2% NDS in PBST for 5 × 15 minutes and incubated in secondary antibodies (donkey anti-rabbit 594 and/or donkey anti-goat 488, 1:200 in blocking solution) for 3 hours at room temperature. Tissues were then washed in PBST 5 times (15 minutes each), mounted on slides with ProLong Gold Antifade Mounting Media (Thermo Fisher Scientific), and imaged using an Olympus VS200 slide scanner (Evident Scientific) and ZEISS 800 upright confocal microscope (Hooker Imaging Core, UNC Chapel Hill). Embryonic whole-mount immunofluorescence images were analyzed by 2 observers using ImageJ (NIH) under a blinded protocol, which identified vessel length, vessel width, vessel branches, and distance between vessel fronts.

### Cell culture.

Male primary human dermal LECs (PromoCell, C-12216, lot numbers 483Z001-2, 489Z014, 493Z030, 506Z015, 500Z019) were cultured in an Endothelial Cell Growth medium MV2 (PromoCell, C-22121) at 37°C under 5% CO_2_. Experiments were done within 5 passages to preserve lymphatic identity. Biological replicates were considered as independent experiments performed with cells cultured on different dishes on different days.

### siRNA knockdown of ARRB1 and ARRB2 gene in LECs.

siRNA against ARRB1 or -2 and a scramble control siRNA were synthesized by Sigma-Aldrich as previously described (65). The siRNA sequences targeting β-arrestin 1 (NM_020251) and β-arrestin 2 (NM_004313) were 5′-AAAGCCUUCUGCGCGGAGAAU-3′ and 5′-AAGGACCGCAAAGUGUUUGUG-3′. The siRNAs were transfected with Lipofectamine RNAiMax (Invitrogen) according to the manufacturer’s instructions. Briefly, LECs were plated and allowed to grow for 24 hours. Cells were transfected with Lipofectamine RNAiMAX reagent (Thermo Fisher Scientific), with fresh media replaced the following day. To confirm knockdown, RNA was purified with TRIzol reagent (Invitrogen, Thermo Fisher Scientific), and mRNA expression was determined using qRT-PCR. The RNA concentration was measured with a NanoDrop Spectrophotometer (Thermo Fisher Scientific). Cells transfected with siRNA were also harvested with urea-based protein lysis buffer for Western blots.

### Quantitative reverse transcription PCR.

cDNA was synthesized using M-MLV reverse transcriptase (Thermo Fisher Scientific, 28-025-013). Quantitative gene expression was assessed using the TaqMan gene expression paradigm (Applied Biosystems), and PCR was performed using a Quant Studio 7 Flex Real-Time PCR System (Applied Biosystems). The following Thermo Fisher TaqMan gene expression assay probes were used for real-time PCR (qPCR): *ARRB1*: Hs00244527_m1; *ARRB2*: Hs00244527_m1; *CALCRL*: Hs00907738_m1; *ACKR3*: Hs00664172_s1; *GPRC5B*: Hs00212116_m1; and *APLNR*: Hs00270873_s1. Relative expression levels were determined with the *ΔΔCt* method and normalized to the reference gene expression of GAPDH.

### EdU cell proliferation assay.

After 4 hours of serum starvation, LECs cultured on glass coverslips and transfected with *siSCR*, *siARRB1*, *siARRB2*, or *siARRB1/2* were incubated with 10 μM EdU (5-ethynyl-2′-deoxyuridine) for 6 hours. EdU staining was performed based on the manufacturer’s instructions of the Click-iT EdU Cell Proliferation kit for imaging (Thermo Fisher Scientific, C10339). The percentage of the EdU-positive cell ratio was determined by the number of EdU-positive nuclei divided by the number of Hoechst-positive nuclei.

### Plasma membrane protein isolation.

Plasma membrane isolation was performed as previously described (66, 67). Briefly, after appropriate treatment, cells were washed with ice-cold 1× PBS and scraped into a tube. While on ice, cells were homogenized by passing 10 times through an insulin syringe (29G1/2). Homogenized cells were spun at 1,000*g* for 10 minutes at 4°C. The supernatant was collected and spun again at 14,000*g* for 20 minutes at 4°C. The supernatant was aspirated, and the remaining pellet, composed of the membrane fraction, was either stored at –80°C or immediately lysed in lysis buffer and rotated for 30 minutes at 4°C before protein normalization.

### Western blot.

First, 1.2 × 10^5^ LECs per well were seeded into 6-well plates. After cells reached 50%–60% confluence, media were replaced with Opti-MEM (Gibco, 31985070) for 4 hours of serum starving. Then, LECs were transfected with either siSCR, siARRB1, siARRB2, or siARRB1 and siARRB2 and cultured for 48 hours with Opti-MEM. LECs were treated with or without 100 nM human AM (Bachem, 4030215.1000) for 4 hours and washed with PBS twice and harvested with urea lysis buffer (50 mM Tris-HCl pH 7.4, 150 mM NaCl, 1% NP-40, 0.25% deoxycholate, 1 mM EDTA, 2 M urea) supplemented with protease (cOmplete EDTA-free Protease Inhibitor Cocktail; Roche, 11873580001) and phosphatase (PhosSTOP EASYpack; Roche, 4906845001) inhibitors as well as Benzonase nuclease (MilliporeSigma, E1014). Protein levels were determined by Pierce BCA protein assay (Thermo Fisher Scientific, 23225). Cell lysates were normalized to the same concentrations by mixing with urea lysis buffer, 4× LDS buffer (Invitrogen, NP0007), and 1 M DTT, and boiled for 10 minutes at 95°C. Normalized cell lysates were then separated on a 4%–12% NuPAGE Bis-Tris gel (Thermo Fisher Scientific, NPO335BOX or NP0336BOX) using 1× MOPS-SDS running buffer (Boston BioProducts, BP-178), transferred to a nitrocellulose membrane using 2× Bis-Tris Transfer buffer (Boston BioProducts, BP-193) supplemented with 20% methanol, and imaged on a Li-Cor Odyssey CLx using Image Studio v5.2. Densitometry was performed using ImageJ software (v1.52a). Two gels were run for each sample, one to probe for phosphorylation of ERK, AKT, and CREB, and the other gel for total protein levels. GAPDH served as a loading control. The following antibodies were used in the Western blot assay: p-p44/42 MAPK (T202/Y204) (Cell Signaling Technology, 4370, 1:5,000), p44/42 MAPK (ERK1/2) (Cell Signaling Technology, 9102, 1:5,000), pAKT(S473) (Cell Signaling Technology, 4060, 1:5,000), AKT(pan) (Cell Signaling Technology, 4691, 1:5,000), P-CREB(Ser133) (Cell Signaling Technology, 9198, 1:5,000), CREB(Cell Signaling Technology, 9197, 1:5,000), VE-cadherin (R&D Systems, AF1002, 1:5,000), β-catenin (BD Biosciences, 610153, 1:5,000), GAPDH (Novus Biologicals, NB300-221, 1:10,000), goat anti-rabbit IR Dye 800 CW (Li-Cor, 926-32211, 1:10,000 dilution), goat anti-mouse IR dye 800 CW (Li-Cor, 926-32210, 1:10,000 dilution), goat anti-mouse IR dye 680 CW (Li-Cor, 926-68020, 1:10,000 dilution).

### LEC Transwell in vitro permeability assay.

LECs (1.2 × 10^5^ per well) were seeded into 6-well plates. After reaching 50%–60% confluence, LECs were transfected with siSCR, siARRB1, siARRB2, or siARRB1 and siARRB2. After 24 hours of transfection, 20,000 LECs were seeded on a Transwell insert (Corning, 0.4 μm pore size) of a 24-well plate. The Transwell inserts were pretreated with human fibronectin (Thermo Fisher Scientific, 5 μg/mL) for 1 hour at room temperature. After reaching confluence, LECs were serum starved in Opti-MEM for 4 hours. Next, 500 μL red-phenol-free DMEM was added to new blank 24-well plates, and media in the inserts were replaced with 200 μL of FITC-dextran (Sigma-Aldrich, 2,000 kDa, 100 μg/mL) in red-phenol-free DMEM. LECs were then incubated at RT for 20 minutes and protected from light. Next, 100 μL of the FITC-dextran suspension media of the lower well was collected, and the fluorescence intensity was measured on the Cytation5 using 490/520 nm excitation/emission in 96-well plates. Four technical replicates were measured, and 4 biological replicates were used per condition in each experiment. The fluorescence intensity in each group was normalized to the average of control wells (siSCR) in each experiment.

### Fluorescent immunostaining and lymphatic junctional assay in LECs.

After 4 hours of serum starvation, LECs cultured on glass cover slips and transfected with either siSCR, *siARRB1*, *siARRB2*, or siARRB1 and siARRB2 were incubated with 100 nM AM or vehicle separately for 20 minutes, and then cells were washed with cold PBS 3 times and fixed with 4% PFA for 30 minutes at room temperature. LECs were then rinsed with PBS 3 times and permeabilized in 0.2% Triton X-100 for 2 minutes followed by 2 washes with PBS. LECs were blocked with 5% NDS in PBS for 2 hours at room temperature and incubated with rabbit anti-VE-cadherin antibody (1:200, Abcam, ab33168) overnight at 4°C. LECs were then washed with 5% NDS in PBS 3 times and incubated with secondary antibody (AffiniPure donkey anti-rabbit 594, 1:500 in 5% NDS in PBS) and for 2 hours at room temperature. After 1 wash with 5% NDS in PBS, LECs were incubated with Hoechst 33258 (1:1,000, Sigma Aldrich) for 10 minutes. LECs were then washed 3 times with 3% BSA in PBS and 1 time with PBS. LECs were then mounted with Prolong Diamond Antifade Mountant (Thermo Fisher Scientific, P36961). Cells were imaged using a ZEISS 800 confocal microscope. The relative membrane and perinuclear VE-cadherin intensities were quantified using Fiji software. Junction types were also quantified using Fiji. Junctional morphology was defined as either continuous (zipper) or discontinuous (button). Continuous (zipper) junctions were defined as cell borders in which more than 75% of the junction length displayed linear, uninterrupted, convex border segments. Discontinuous (button) junctions were defined as borders in which more than 75% of the junction length exhibited concave patterning. Multiple fields of view from the same biological replicate and 3 biological replicates were used for quantification.

### Statistics.

All normalized in vivo data analysis was performed on a per-litter basis. Briefly, the mean value of control embryos was calculated within each litter, and all values from each group within that litter were normalized to the mean of the control group. Statistical analyses were performed using GraphPad Prism version 9.4.1. Data normality was evaluated along with a 1-way ANOVA or 2-tailed unpaired Student’s *t* test to compare groups. Summary data are presented as average values with standard deviation. *P* values less than 0.05 were considered significant. The statistical test performed is noted in corresponding figure legends.

### Study approval.

All animal studies were approved by the IACUC of UNC Chapel Hill. Animal housing, care, and husbandry were overseen by the UNC Division of Comparative Medicine Animal Resources, which is accredited by the Association for Assessment and Accreditation of Laboratory Animal Care.

### Data availability.

All data in the manuscript are included in the Supporting Data Values file.

## Author contributions

KMC and YT conceived and designed research. YT, DSS, ESD, MAZ, and DMD performed experiments. YT, DSS, MAZ, AMT, BMK, and NMTV analyzed data. YT, DSS, MAZ, and KMC interpreted the results of experiments. YT prepared figures and drafted the manuscript. YT, DSS, AMT, MAZ, ESD, and KMC edited and revised the manuscript. YT, DSS, AMT, ESD, KMC, BMK, NMTV, DMD, and MAZ approved the final version of the manuscript.

## Conflict of interest

The authors have declared that no conflict of interest exists.

## Funding support

This work is the result of NIH funding, in whole or in part, and is subject to the NIH Public Access Policy. Through acceptance of this federal funding, the NIH has been given a right to make the work publicly available in PubMed Central.

- This study was supported in part by P30 CA016086 Cancer Center Core Support grant to the UNC Lineberger Comprehensive Cancer Center.
- UNC Pathology Services Core is supported in part by a National Cancer Institute Center Core Support grant (P30CA016086).
- NIH grants from the National Heart, Lung, and Blood Institute (NHLBI HL1290986) and the Eunice Kennedy Shriver National Institute of Child Health and Human Development (NICHD HD060860) (to KMC).
- American Physiological Society Postdoctoral Fellowship and American Heart Association Postdoctoral Fellowship 24POST1188946 (to YT).

## Supplementary Material

Supplemental data

Unedited blot and gel images

Supporting data values

## Version 1 03/26/2026

In-Press Preview

## Version 2 05/22/2026

Electronic publication
