# Supplementary material for: β-Arrestin1/2 are essential for embryonic lymphatic vessel development
Source: JCI Insight. 2026 Mar 26;11(10):e198032. doi: 10.1172/jci.insight.198032 (PMC13232720; doi:10.1172/jci.insight.198032)
Supplement: Supplemental data [file jciinsight-11-198032-s009.pdf]

Supplementary Fig 1

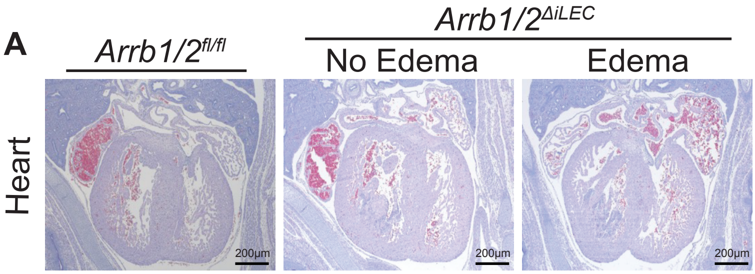

A. H&E staining shows enlarged and blood-filled jugular lymphatic sacs (JLS) of *Arrb1/2*$^{\Delta iLEC}$ embryo at E15.5. CA: Carotid Artery; eJV: external jugular vein; JLS: Jugular Lymphatic Sac. Scale bar: 100μm.

Supplementary Fig 2

A

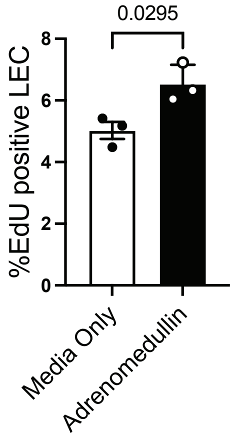

Quantification of EdU positive proliferation of LECs transfected with scramble siRNA (siSCR) and treated with or without adrenomedullin n = 3 biological replicates per group. Unpaired t-test.

Supplementary Fig 3

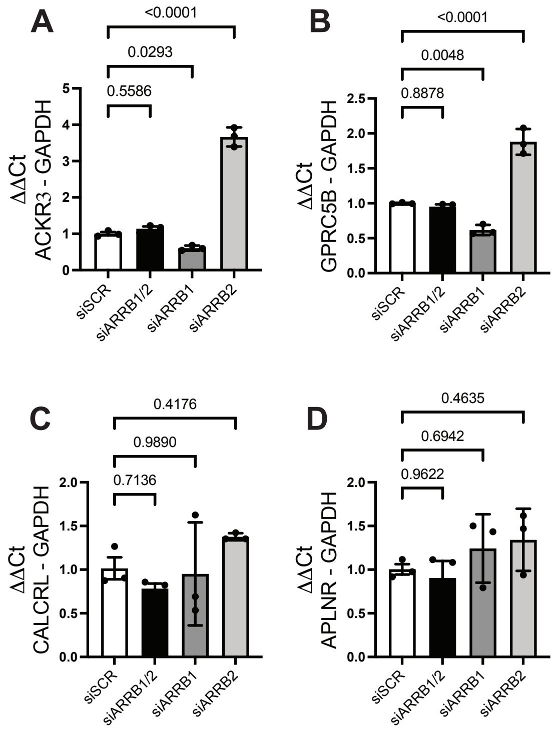

Quantification of ACKR3 (A) and GPRC5B (B), CALCRL (C) and APLNR (D) mRNA expression in LECs transfected with scramble, ARRB1, ARRB2 or ARRB1/2 siRNA. n = 3 biological replicates per group. One-way ANOVA.

Supplementary Fig 4

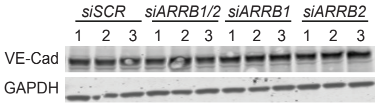

Western-blot show VE-Cadherin and GAPDH expression in LECs transfected with scramble, ARRB1, ARRB2 or ARRB1/2 siRNA. n = 3 biological replicates per group.
