# Supplementary figures and images for: β-Arrestin1/2 are essential for embryonic lymphatic vessel development
Source: JCI Insight. 2026 Mar 26;11(10):e198032. doi: 10.1172/jci.insight.198032 (PMC13232720; doi:10.1172/jci.insight.198032)

Fig. 1A

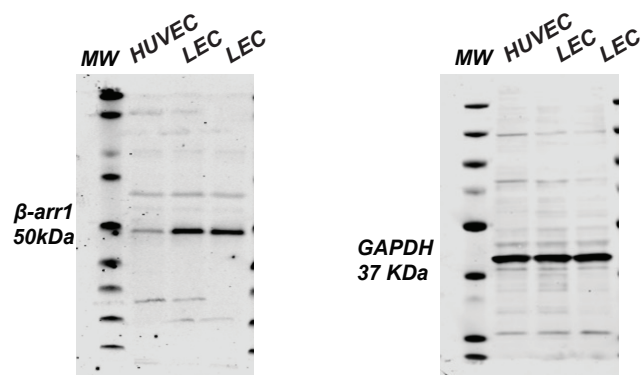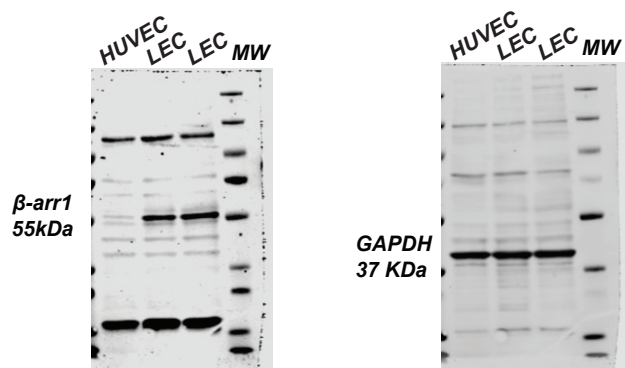

Fig. 2C

SRY genotyping: E15.5, *Arrb1/2<sup>ΔILEC</sup>* embryos

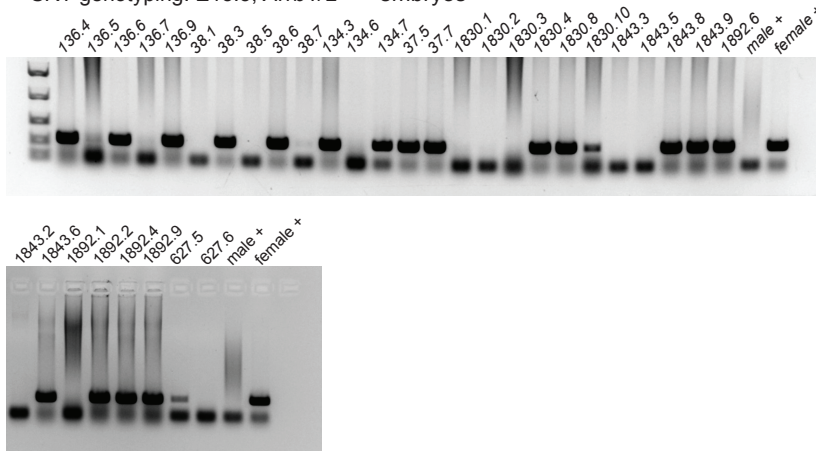

Fig. 4A

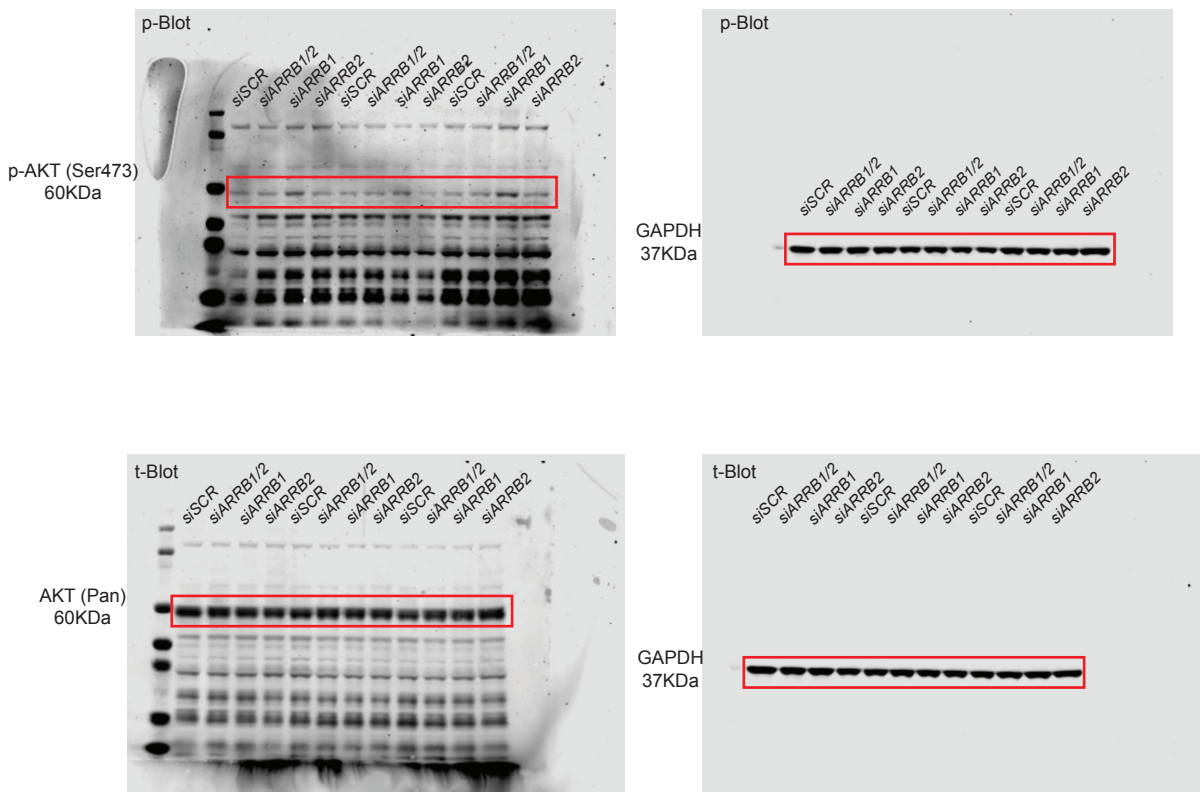

Fig. 4C

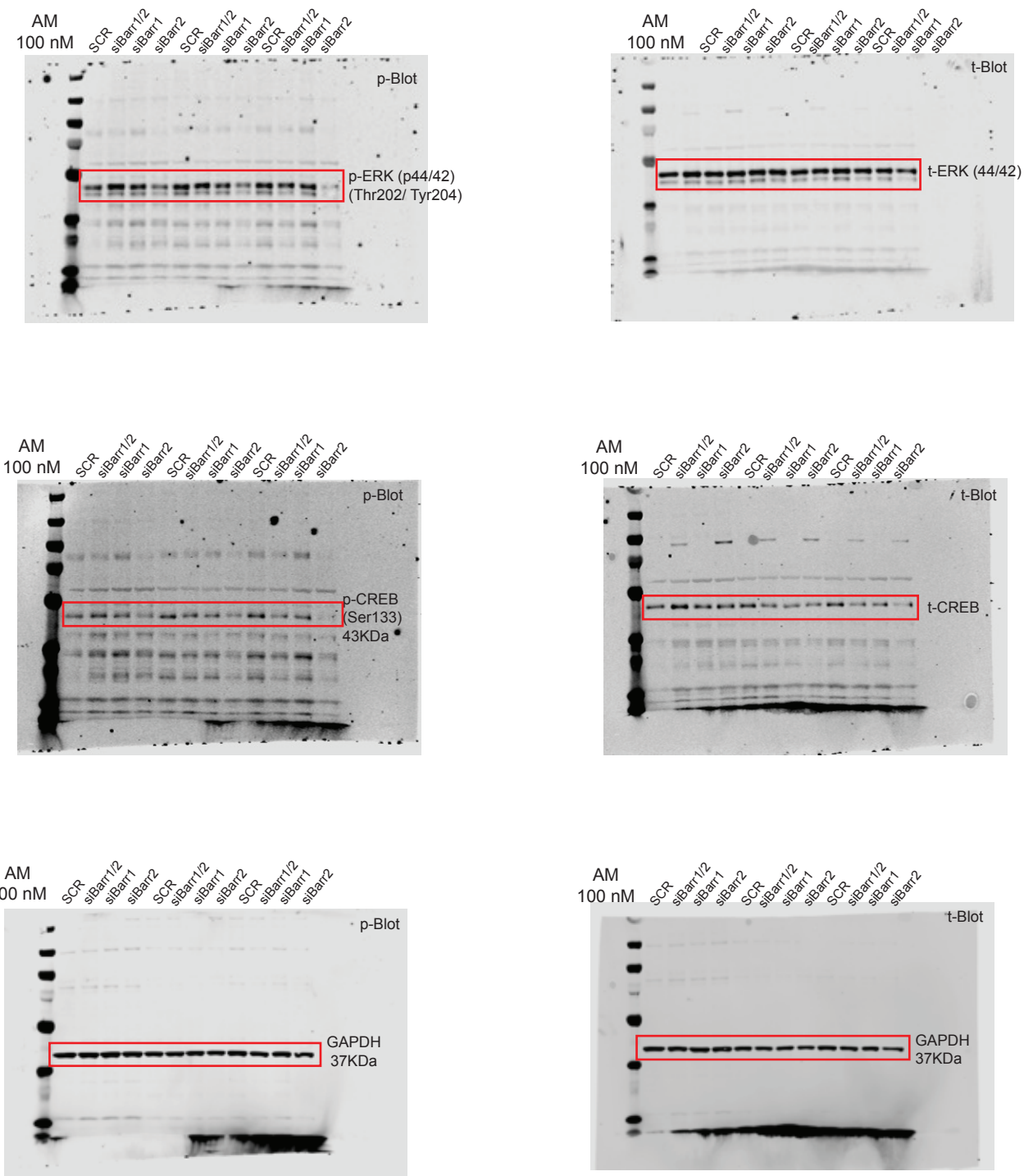

Fig. 6E

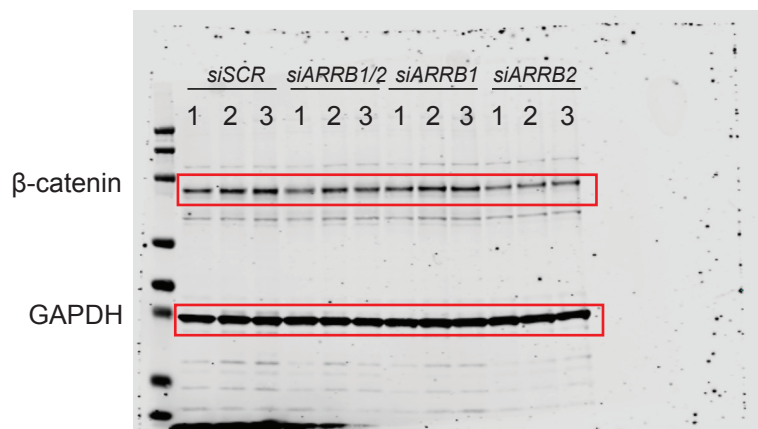

Supplement: Unedited blot and gel images [file jciinsight-11-198032-s010.pdf]
